# Differential Regulation of Rab GTPase Expression in Monocyte-Derived Dendritic Cells upon Lipopolysaccharide Activation: A Correlation to Maturation-Dependent Functional Properties

**DOI:** 10.1371/journal.pone.0073538

**Published:** 2013-09-05

**Authors:** Axel Berg-Larsen, Ole J. B. Landsverk, Cinzia Progida, Tone F. Gregers, Oddmund Bakke

**Affiliations:** Centre for Immune Regulation, Department of Biosciences, University of Oslo, Oslo, Norway; National Institute of Biological Sciences, Beijing, China

## Abstract

The regulation of Rab expression to modulate cellular function has recently been proposed. Dendritic cells are a prototypic example of cells that drastically alter their function in response to environmental cues by reducing endocytosis, secreting cytokines, changing surface protein repertoires and altering morphology and migration. This is not a binary event, but is subject to fluctuations through the activation process, termed maturation. Consequently, DCs transiently increase endocytosis and production of major histocompatibility complex class II molecules, and secrete inflammatory cytokines in infected tissues before migrating to secondary lymph nodes and releasing T cell polarizing factors. All these cellular processes rely on intracellular membrane transport, which is regulated by Rab family GTPases and their diverse effectors. Here we examine how the Rabs likely to be involved in these functions are regulated throughout DC maturation. We find that Rab expression is altered upon lipopolysaccharide-induced activation, and discuss how this correlates to the reported functions of these cells during maturation.

## Introduction

The organization of the complex network of intracellular membrane-enclosed compartments is essential to the homeostasis and function of all eukaryotic cells. Transport and maintenance of this network is largely regulated by the small GTPases from the Rab family. Rabs are reversibly bound to cellular membranes via one or two hydrophobic geranylgeranyl groups and, via the recruitment of various effector proteins, they facilitate cargo selection, membrane motility, fusion and fission [1]. Rab activity is regulated by GDP dissociation inhibitor (GDI) dissociation factors (GDFs), which target Rabs to specific membranes and promote GDI release and geranylgeranyl membrane insertion. Guanine nucleotide exchange factors (GEFs) act on membrane-bound Rabs to release GDP and convert them to their active (GTP-bound) state. GTPase activating proteins (GAPs) enhance the intrinsic GTPase activity of Rabs to revert them to their inactive state in which they are substrates for GDIs, which extract the Rabs from membranes and chaperone geranylgeranylated Rabs in the cytosol. More than 60 Rabs have been identified in humans and these seem to play distinct, non-redundant roles in membrane transport [2].

Dendritic cells (DCs) are professional antigen presenting cells with an exceptional ability to activate naïve T-cells. In the steady state they act as sentinels to detect pathogens and after activation they initiate innate and adaptive immune responses through the secretion of cytokines and presentation of antigenic peptides on major histocompatibility complex molecules (MHC). DCs are therefore considered to exist in two distinct developmental stages, determined by pathogen exposure, with different functional characteristics. Steady state, *immature* DCs (iDCs) are highly adept at taking up antigens by phagocytosis or macropinocytosis and processing internalized antigen. In contrast, mature DCs (mDCs) have a low antigen uptake and processing capacity, but are efficient activators of naïve T cells through the expression of MHC and co-stimulatory molecules, and secretion of cytokines. In between these two developmental stages, DCs transiently increase then decrease endocytic activity and the production of MHC II molecules. Motility is transiently decreased and different cytokine repertoires are secreted to activate innate cells in tissues and direct T cell development in lymph nodes [3]. In addition to the altered capabilities for endocytosis, secretion and migration, DCs exhibit a remarkable morphological restructuring of the lysosomal compartments during maturation, with extensive tubules involved in the focal delivery of MHC II to the DC surface [4,5].

These profound morphological and functional changes during DC maturation clearly require significant changes in the organization and regulation of intracellular trafficking. Here we investigate how the relative mRNA levels of representative Rab GTPases are regulated during DC maturation. We selected 17 Rabs and one Arf based on their reported involvement in endocytosis, endosomal/lysosomal function, secretion/exocytosis and adhesion/migration (Table S1). Our results demonstrate an activation-induced differential regulation of Rab expression in DCs, and we propose that this regulation correlates with the reported altered functional properties of DCs during maturation.

## Results and Discussion

### Rab Proteins Are Expressed at Different Levels in iDCs

While intracellular transport and organelle specialization are essential to the function and homeostasis of all cells, it is clear that cells with particular functions require specific adaptations. Expression of certain Rabs is cell type specific, such as Rab27a, which is expressed in non-neuronal secretory cells conducting regulated exocytosis [6]. Furthermore, the expression level of ubiquitously expressed Rabs varies depending on cell type (Figure S1), indicating that cells maintain the level of Rabs to achieve their particular requirements. The regulation of intracellular transport through transcriptional control is a fairly novel concept, but is supported by a growing body of data [7].

Dendritic cells exhibit a particularly intriguing altered functional behavior in response to pathogenic stimuli, and represent an ideal system to examine how altered function correlates with Rab expression profiles. To investigate the expression of Rabs in DCs during maturation, we generated DCs from monocytes negatively sorted from human blood and cultured for 5-6 days in GM-CSF and IL-4. The resultant cells (moDCs) respond characteristically to activation with the TLR4 ligand lipopolysaccharide (LPS) by increasing expression of MHC II, the co-stimulatory molecule CD83 and the lymph node homing receptor CCR7, as previously described [8].

**Figure 1:**
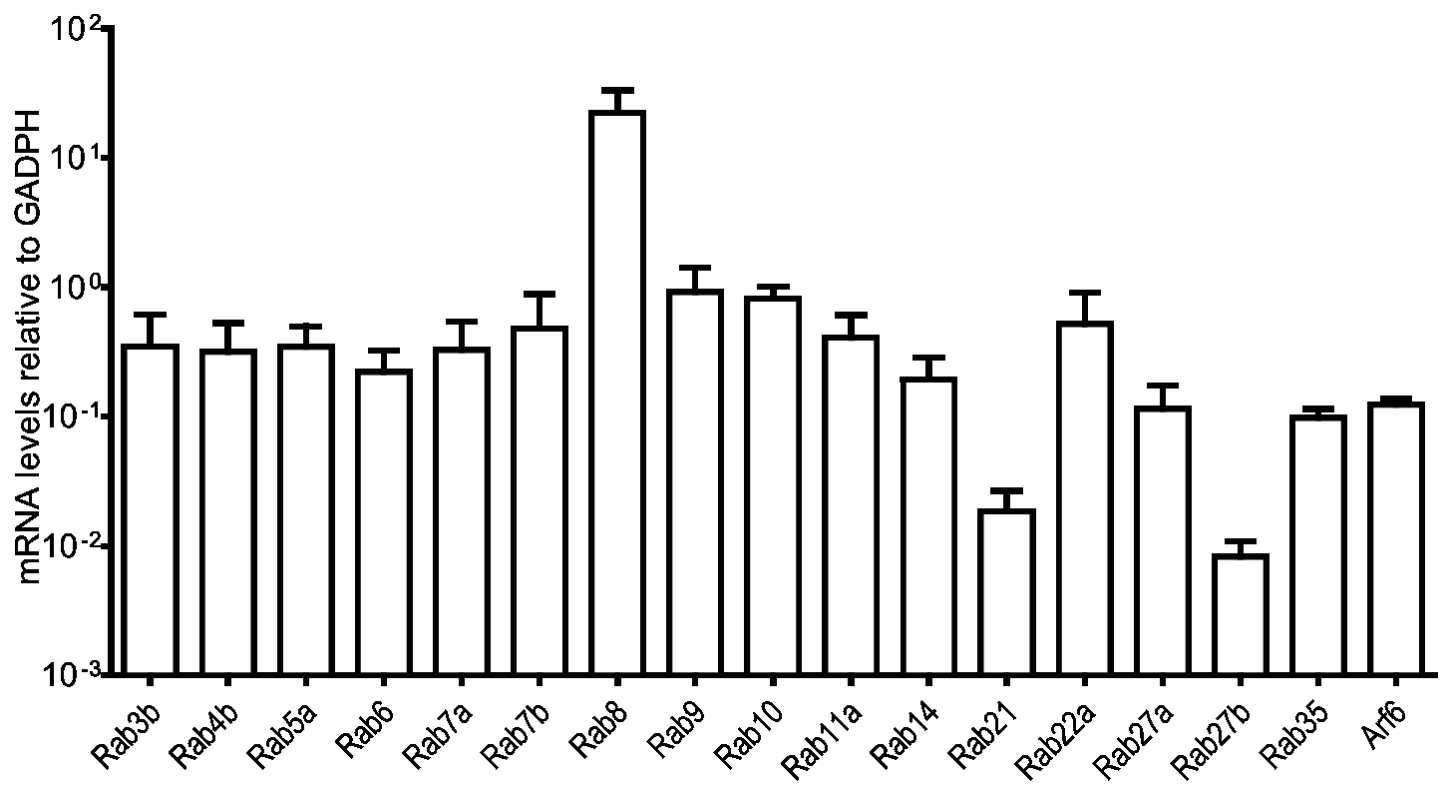
Rabs show large variation in expression levels in iDCs. Quantitative RT-PCR shows expression levels of the targeted Rab GTPases prior to activation of moDCs. Means and SD are shown on a logarithmic scale, n=6. Expression levels have been normalized to the reference gene GADPH.

We first harvested mRNA from iDCs, performed qPCR and measured the basal expression of the Rabs relative to the reference gene Glyceraldehyde 3-phosphate dehydrogenase (GAPDH), which is stably expressed during maturation [8]. As expected, the Rabs exhibit a large variation in expression level. Most highly expressed in iDCs was Rab8a, followed by Rab9, Rab10, Rab22a and Rab7b (Figure 1). Rab7b and Rab9 both function in the recycling of mannose-6-phosphate receptors (M6PRs) from endosomes to the trans-Golgi network (TGN), and consequently make receptors available at the TGN for the transport of hydrolytic enzymes into the endocytic pathway [9,10]. Rab10 and Rab22a are reportedly involved in promoting or delaying phagosomal maturation, respectively [11,12], although Rab10 also plays a role in endoplasmic reticulum (ER) tubule formation [13]. Rab8a, which exhibits a particularly high expression in iDCs, is involved in membrane protrusions leading to macropinocytosis [14], extension of ciliary membranes [15], but also actin-based movement of lysosome-related organelles (melanosomes) [16], and docking and fusion of exocytic vesicles [17]. Membrane extensions and macropinocytosis, degradation of endocytosed cargo, movement of lysosome related organelles containing MHC II and exocytosis are all essential features of DCs [18], indicating that the expression levels of Rabs reflects the functional properties of DCs in the steady state.

To assess whether the expression pattern in iDC was particular for these cells or a general scheme of Rab expression, we compared the mRNA levels in iDCs with those from the human cell lines HeLa and MelJuSo. MelJuSo is a cell line derived from a human melanoma, which due to its endogenous expression of MHC II has been widely used to study MHC II transport, and the validity of findings in this cell line has been correlated and confirmed for human DCs [19]. We found that the expression pattern of Rabs in HeLa and MelJuSo differ greatly from those of iDCs, with a generally lower expression of most Rabs relative to GAPDH, and no correlation between the expression ratios of the individual Rabs (Figure S1). Thus, the particular Rab expression pattern in iDCs indicates a requirement for specific Rab expression levels to achieve the functional requirement of iDCs. Next, we analyze how the expression of individual Rabs is modulated during DC maturation, using the expression levels in iDCs (Figure 1) to normalize mRNA measured 4, 8, 24 and 48 hours after LPS stimulation.

### Endocytic Rabs are increased at early time points after LPS stimulation

Although the role of Rabs in various forms of endocytosis is not entirely clear, several reports suggest that Rabs are involved in regulating membrane dynamics leading up to phagocytosis and macropinocytosis. A common feature of DC maturation is the transient boost in macropinocytosis and phagocytosis followed by a more or less complete arrest [20,21]. Both endocytic mechanisms involve actin polymerization directed by Rho family GTPases and delivery of membranes through focal exocytosis of recycling endosomes [22]. Rab5, Rab8a and Rab35 are all reportedly involved in facilitating actin remodeling during macropinocytosis and phagocytosis through recruitment and activation of the Rho-family GTPases Cdc42 and Rac1 [14,23–26]. These Rabs exert their effects via the non-Rab small GTPase Arf6, which coordinates Rac1 activation in membrane protrusive events [27,28]. We included Rab21, which also localizes to macropinosomes in macrophages, albeit at a later stage than Rab5 [29], but shares the Rab5 GEFs Rabex-5 and VPS9-ankyrin-repeat protein (Varp) [30,31], and the macropinosome localized effector APPL1 [32,33].

**Figure 2:**
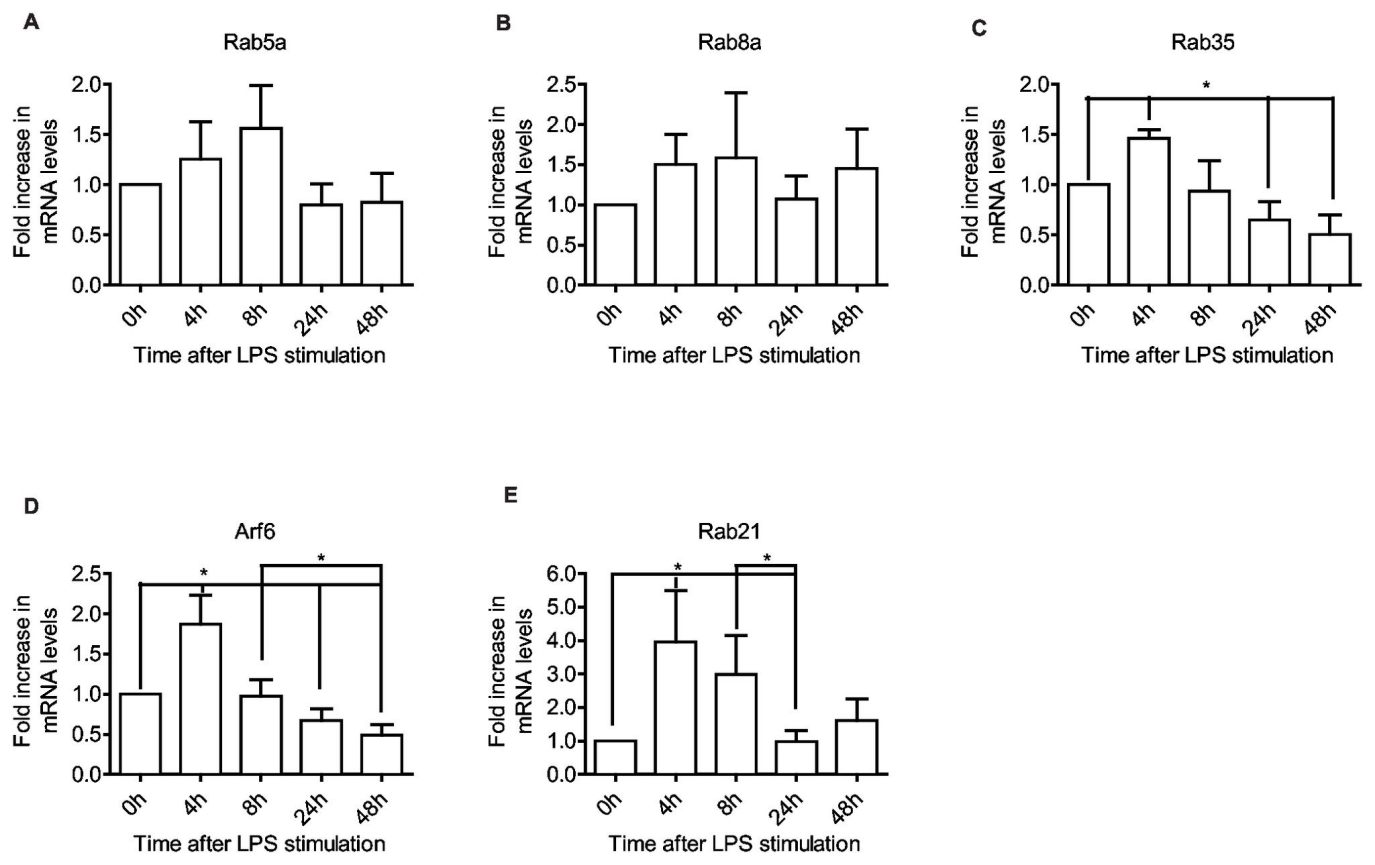
Rabs involved in endocytosis exhibit an early increase after LPS activation. Quantitative RT-PCR shows expression levels of Rab5a (A), Rab8a (B), Rab35 (C), Arf6 (D) and Rab21 (E) in moDCs, after LPS stimulation for the indicated time periods. All data are normalized to initial iDC expression levels. Means and SD are shown, n = 6, * P<0.05.

Rab5 exists in three isoforms (a, b, c) with high homology and similar function in endocytosis [34], of which Rab5a is the best characterized. Rab5a and Rab8a exhibit an increase up to 8 hours after TLR4 activation, then return to initial levels by 24 hours (Figure 2, A-B). Rab35 and Arf6, however, peak after 4 hours then decrease to initial levels after 8 hours and gradually to below initial levels 24 and 48 hours after activation (Figure 2, C–D). Rab21 exhibits a particularly strong response, up 4-fold after 4 hours, which then decreases gradually to the initial level after 24 hours before rebounding slightly after 48 hours (Figure 2, E). The stronger response of Rab35 and Arf6 compared to Rab5a and Rab8a may reflect a more immediate link to actin polymerization. Rab35 has been shown to recruit ACAP2 [26], which is a GAP for Arf6, and Arf6 is required for targeting Rac to the plasma membrane [35]. Although Rab5 apparently facilitates the activation of Rac by delivering it to endosomes containing the Rac GEF Tiam1 [24], the slower response and sustained levels of Rab5a and Rab8a may be due to a more general requirement for these Rabs in DC function. Indeed, Rab5 is required for the biogenesis and maintenance of the endo-lysosomal system in all cells [36], and although Rab8a has been linked to Arf6 and membrane ruffling events [14], it is also involved in the actin-dependent movement of lysosome-related organelles [16] and in the docking and fusion of secretory vesicles [37]. The very strong early increase in Rab21 is interesting. Although Rab21 has been detected on macropinosomes it, unlike dominant negative Rab5, was not found to affect the rate of macropinosome formation [29,38]. A dominant negative mutant of Rab21 has been shown to decrease receptor-mediated endocytosis [39], and this mechanism is maintained by DCs after activation [40]. However, Rab21, Arf6 and Rab35 are also involved in integrin transport leading to membrane protrusion, adhesion and cell migration [41–47], and since moDCs tend to extend dendrites and adhere more tightly early after activation (Figure S2), the increase may be involved also in this aspect of DC maturation.

### Expression levels of Rabs involved in endosomal recycling increase in the early phase of DC maturation

**Figure 3:**
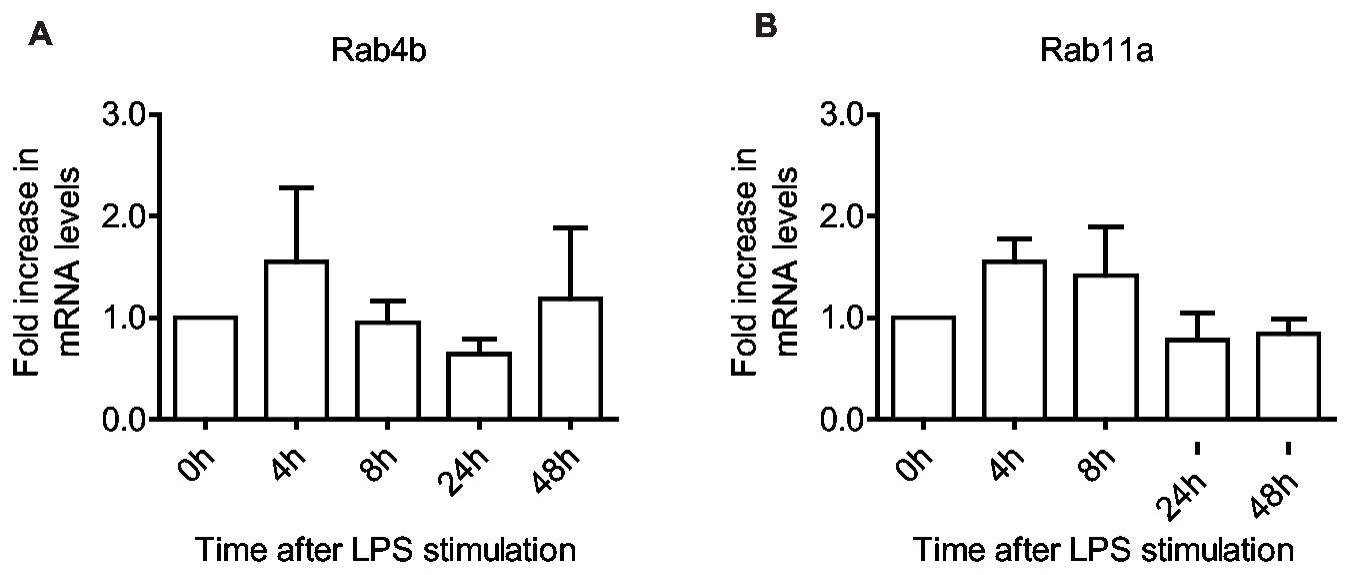
Endosomal recycling Rabs increase in the early phase of DC maturation. Quantitative RT-PCR shows expression levels of the targeted Rab GTPases in moDCs, after LPS stimulation for the indicated time periods. All data are normalized to initial iDC expression levels. Rab4b (A) and Rab11a (B) are involved in short and long loop recycling, respectively. Means and SD are shown, n = 6.

Recycling of membrane and receptors is an important mechanism not only in maintaining the level of cell surface proteins such as MHC II [48], but also in cell adhesion and protrusion [49], migration [50] and phagocytosis [51]. Rab4 and Rab11 are required for short and long loop recycling of cargo to the cell surface, respectively [52]. Rab4 and Rab11 are ubiquitously expressed, whereas the expression of Rab4b is controlled by the class II trans-activator and consequently restricted to MHC II expressing cells [53], although its distribution and function appears to be similar [54,55]. We find that as expected Rab4b follows a similar pattern of regulation to MHC II [8], with an early increase at 4 hours followed by a decrease after 8 and 24 hours (Figure 3 A). Surprisingly, the level of Rab4b returns to initial levels 48 hours after LPS stimulation, thus possibly, as we observed for invariant chain [8], the regulation of Rab4b expression is to some extent unlinked from that of MHC II. For Rab11a, the expression level is somewhat similar to Rab5a, with a 50% increase within 4-8 hours followed by a return to initial levels (Figure 3 B). As Arf6, Rab8a and Rab35 have also been shown to function in recycling [45,56,57], and the expression of these Rabs exhibit a similar pattern (Figure 2 B–D), this indicates that elevated expression of recycling Rabs might facilitate the transient increase in macropinocytosis and phagocytosis early after DC activation.

### Late endosomal Rabs respond differentially to LPS stimulation

**Figure 4:**
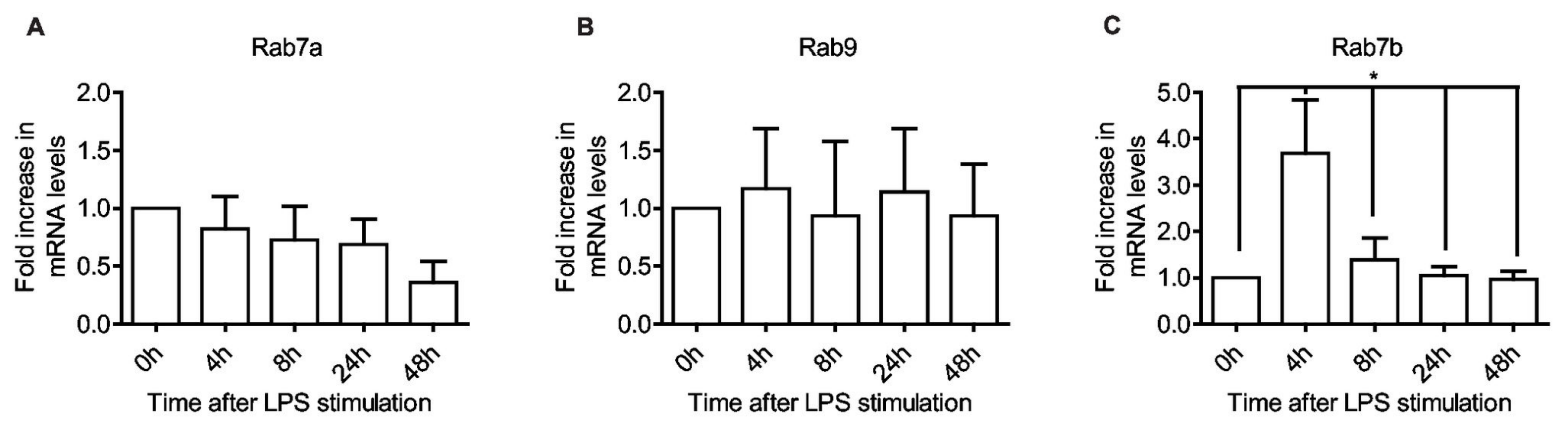
Late endocytic Rabs are differently expressed during DCs maturation. Quantitative RT-PCR shows expression levels of the targeted Rab GTPases in moDCs, after LPS stimulation for the indicated time periods. All data are normalized to initial iDC expression level. Rab7a (A) and Rab9 (B) show weak responses to DC activation compared to Rab7b (C). Means and SD are shown, n = 6, *P<0.05.

Early endosomes, phagosomes and macropinosomes eventually acquire hydrolytic enzymes for digesting engulfed particles in a process generally termed endosomal maturation. Endosome and phagosome maturation involves Rab7a, via its GEF HOPS (*ho*motypic *f*usion and *p*rotein *s*orting complex) and Mon1 [58]. The transport of hydrolytic enzymes from the biosynthetic pathway requires binding to sorting receptors at the TGN, whose recycling back to the Golgi apparatus is regulated by Rab9 and Rab7b [10,59]. Rab7a seems to be only slightly decreased and Rab9 does not exhibit any significant change in expression during maturation. Rab7b, however, increases 4-fold after only 4 hours, and then rapidly declines to initial levels (Figure 4A–C). This pronounced response by Rab7b and not Rab9 might indicate a different specificity for sorting receptors and their cargo by Rab9 and Rab7b. Dominant negative variants or depletion of either Rab impairs Cathepsin D maturation, showing that the recycling of M6PRs back to the Golgi is essential for efficient delivery of proteases to lysosomes [59,60]. However, a dominant positive mutant of Rab7b has a more pronounced effect on the sorting of sortilin than dominant positive Rab9 [60]. Rab9 is ubiquitously expressed whilst the expression of Rab7b is very low except for in a few tissues, most notably cells of monocytic lineages [61], suggesting a more specialized role immune cells. Rab7b has also been implicated in the down-regulation of TLR4 and TLR9 and serves to prevent excessive signaling after TLR ligation [62,63]. Interestingly, in macrophages both TLR4 and TLR9 stimulation lead to an altered Rab7b expression, however in these cells the expression profile of Rab7b is completely inverted. Both TLR4 and TLR9 ligation lead to a decrease in Rab7b expression within 4 hours, which then increases 8-24 hours after activation [62,63]. Thus, in macrophages, which generally have a more aggressive proteolytic endocytic pathway compared to DCs [64], the lower expression of Rab7b should reduce the retrieval of sorting receptors to the Golgi. This could consequently affect the endosomal protease repertoire, possibly to optimize the generation of peptides for loading on MHC. In DCs, which have a less aggressive endocytic environment in the steady state, the increased expression of Rab7b might serve to elevate the repertoire of endosomal proteases in order to enhance proteolysis of antigen and increase the diversity of peptides.

### Rabs involved in delaying phagosomal maturation and degradation are differentially expressed during DC maturation

Phagosomal maturation can be prevented by Rab14 and Rab22a [11,65], whilst Rab27a has been shown to reduce phagosomal degradation [66]. Rab14 is involved in the biosynthetic-recycling pathway between the Golgi and endosomal compartments [67], but also promotes endocytic recycling at an intermediate step between Rab4 and Rab11 [68,69]. Active Rab22 prevents recruitment of Rab7 [11], possibly by recruiting Rabex-5 (a Rab5 GEF) to maintain Rab5 in a GTP-bound state [30,70]. However, Rab22 also induces the formation of tubular recycling endosomes, which are necessary for endosome to cell surface recycling of internalized materials [71]. Rab27a has been shown to play a role in the recruitment of the "inhibitory lysosome-related organelles" containing the NADPH oxidase NOX2 to phagosomes in murine bone-marrow derived DCs, thus leading to reduced antigen degradation and increased cross-presentation on MHC I [66].

**Figure 5:**
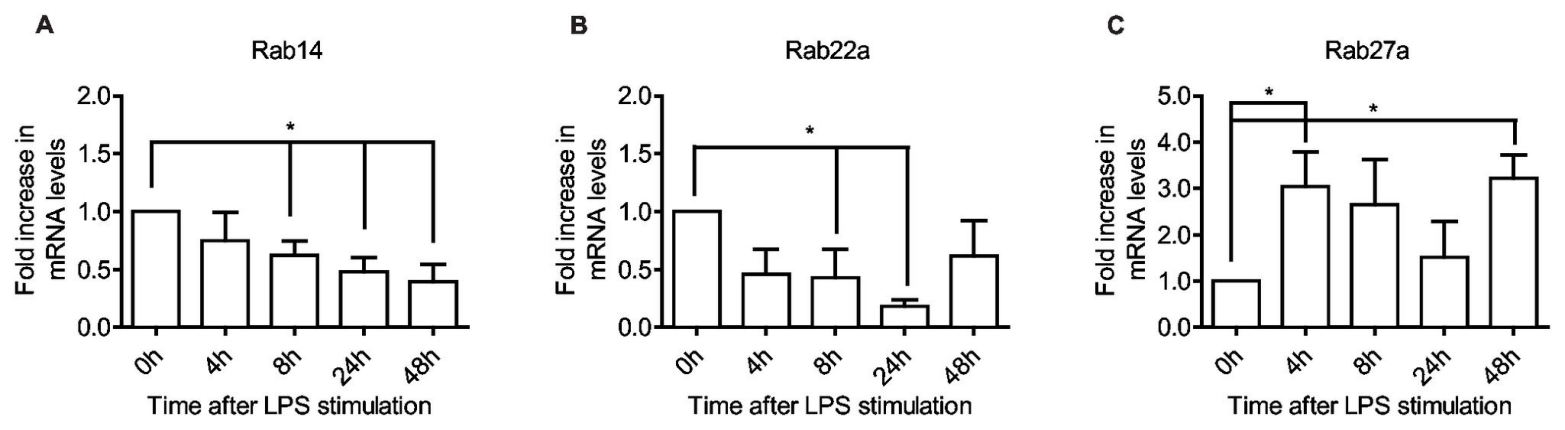
Rabs involved in delaying phagosomal maturation are differentially regulated during the maturation process. Quantitative RT-PCR shows expression levels of Rab14 (A), Rab22a (B) and Rab27a (C) in moDCs, after LPS stimulation for the indicated time periods. All data are normalized to initial iDC expression level. Means and SD are shown, n = 6. *P<0.05, **P<0.01.

We find that Rab14 mRNA is gradually reduced after maturation, whereas Rab22a expression drops more rapidly down to 50% within 4-8 hours, is almost completely absent at 24 hours, then recovers to about 60% of initial levels 48 hours after activation (Figure 5A-B). This early decrease could serve to complement the effects of increased transport of hydrolytic enzymes facilitated by the transiently elevated Rab7b and serve to modulate endosomal proteolysis which has been reported for mature DCs [72]. Rab27a however, which is relatively low in immature cells (Figure 1), increases 3-fold after only 4 hours of LPS stimulation (Figure 5C), thus seemingly counteracting the effect of both increased Rab7b, and decreased Rab14 and Rab22a. Possibly the effect of these Rabs are restricted to subsets of phagosomes, some involving Rab7b facilitating direct presentation on MHC II, others involving Rab27a on MHC I cross-presentation. Such alternative routing of antigen has been described for endocytosis via the mannose receptor leading to cross-presentation and pinocytosis leading to direct presentation on MHC II [73]. Alternatively, moDCs may have different Rab27a effector repertoires from bone-marrow derived DCs and thereby function in other pathways. However, other studies on Rab27a have found that it is involved in regulated exocytosis, such as the release of lytic granules by T cells, NK cells and neutrophils, and melanin by melanocytes [74].

### Rabs involved in exocytosis are differentially regulated after LPS stimulation

**Figure 6:**
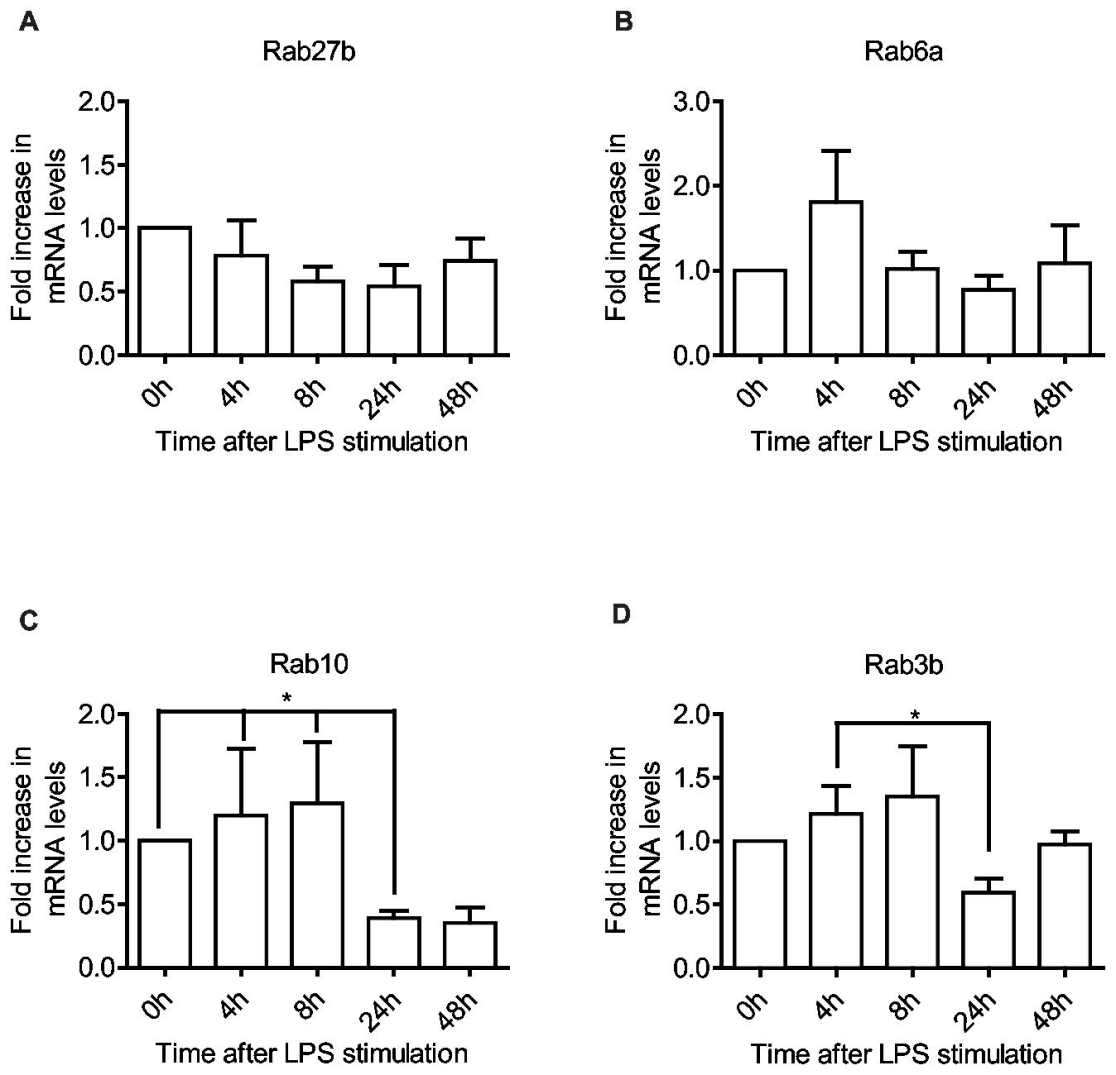
Rab GTPases involved in exocytosis **alter their expression as the DC matures**. Quantitative RT-PCR shows expression levels of Rab27b (A), Rab6a (B), Rab10 (C) and Rab3b (D) in moDCs, after LPS stimulation for the indicated time periods. All data are normalized to initial iDC expression levels. Means and SD are shown, n = 6, *P<0.05.

Secretion of cytokines and exosomes is certainly an important feature of DCs, but the type of cytokine and quantity of the exosomes released is altered as DCs mature [75–77]. Rab3b, Rab6a, Rab8a, Rab10, Rab27a/b and Rab35 have all been implicated in secretion/exocytosis. We find that these Rabs are all increased to varying degrees after activation, with the notable exception of Rab27b (Figure 6 A–D). Rab27b is involved in the delivery of secretory granules to the cell surface and is widely expressed in exocytic cells, but also in cells involved in surface protection and mechanical extension [78,79]. In moDCs, however, Rab27b has a very low expression level in immature cells and this decreases even further during maturation (Figure 1 and Figure 6A), indicating that it does not play a vital role in moDCs in the steady state or during maturation. Apart from Rab27a (Figure 5C), Rab6a expression exhibits the largest increase early after moDC activation, up 2-fold after 4 hours then decreasing to initial levels at 8 hours where it remains for the duration (Figure 6B). Rab10 is slightly elevated 4 and 8 hours after activation and then drops sharply to circa 40% of initial values by 24 hours (Figure 6C). Rab3b closely mirrors the expression of Rab8a (Figure 2B), increasing by 50% within 8 hours, down at 24 hours and recovering at 48 hours (Figure 6D). Rab6a seems to function in the generation and transport of secretory vesicles from the Golgi network towards the cell periphery, where Rab8a facilitates their docking and fusion with the plasma membrane [17,80]. This suggests that the increase in Rab6a could promote the release of inflammatory cytokines early after DC activation [75]. In macrophages Rab10 expression is induced upon LPS stimulation, albeit with slower kinetics than in DCs, and serves to direct the transport of newly synthesized TLR4 from the Golgi to the cell surface and promote the production of inflammatory cytokines [81]. Rab10 has been shown to interact with Myosin-Va, and in adipocytes this facilitates the translocation to, and subsequent docking of glucose transporter isoform 4 (GLUT4) storage vesicles at the plasma membrane [82]. Seemingly then Rab10 plays a role in the biosynthetic pathway for new surface receptors and cytokines, and the controlled release from storage vesicles. However, Rab10 may also influence phagosome maturation as knockdown or expression of a dominant negative mutant was shown to delay phagosomal maturation [12]. Knockdown of Rab3b inhibits calcium-dependent exocytosis in anterior pituitary cells [83], but in a murine DC line (DC2.4-B3Z) it also negatively affects MHC I cross-presentation. It is also increased in macrophages during inflammation and may function with Rab37 in the release of TNF-α [84]. In endothelial cells it was found to be involved in the exocytosis of Weibel-Palade bodies (WPB) [85]. WPBs are specialized lysosome-related organelles similar to the MHC II containing compartments in DCs, and in maturing DCs, these compartments exhibit a dynamic behavior with tubular extensions delivering MHC II to the cell surface [4,86], indicating that elevated Rab3b might also contribute to the increase in surface MHC II or the release of immunogenic exosomes.

### Rab protein levels and activity during DC maturation

**Figure 7:**
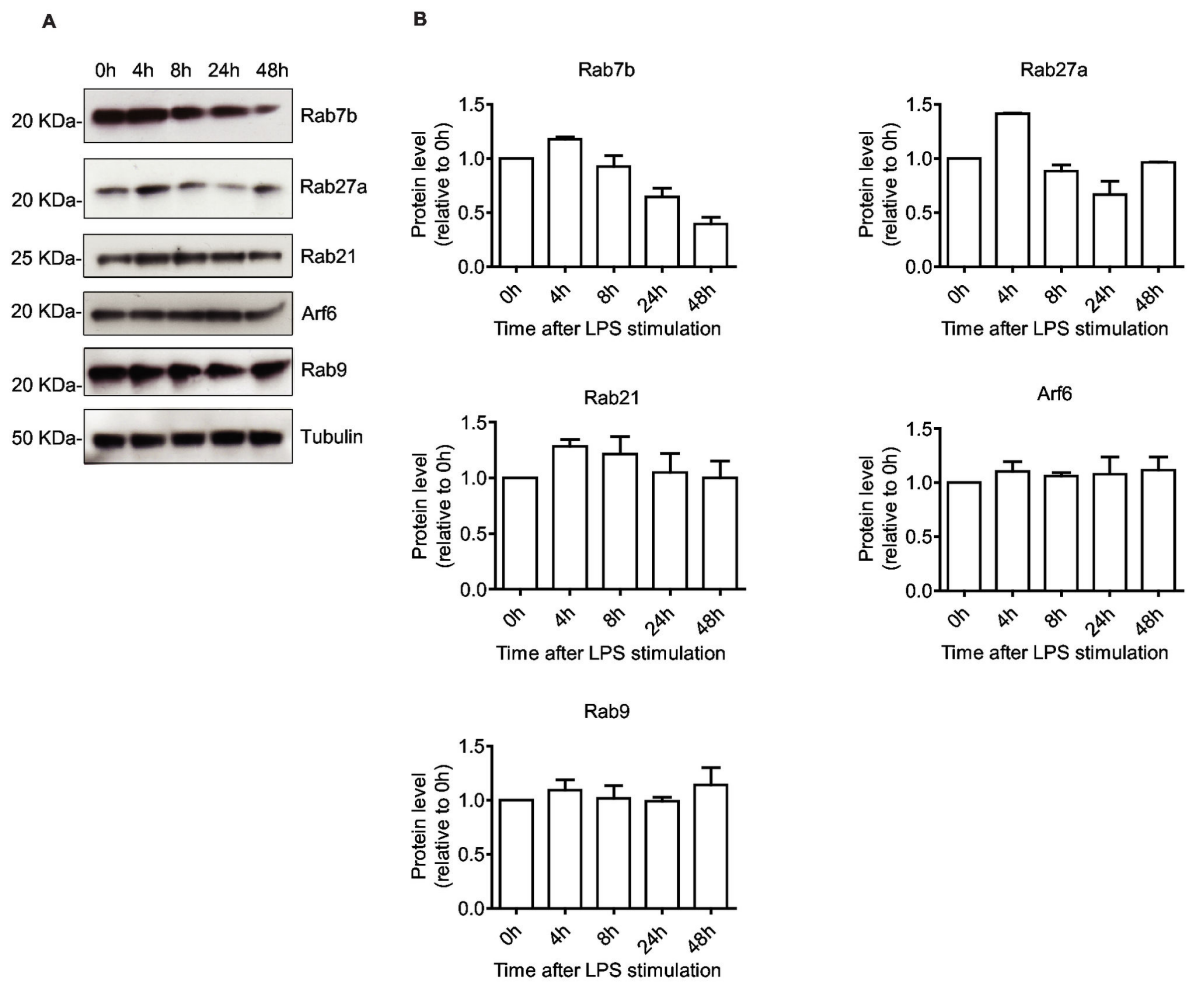
Protein levels of GTPases during DC maturation. (A) Protein lysate from moDCs stimulated with LPS for the indicated time points were run on SDS-PAGE and subjected to Western blot analysis using anti-Rab7b, anti-Rab27a, anti-Rab21, anti-Arf6, anti-Rab9 and anti-tubulin antibodies. (B) The intensity of the bands was quantified by densitometry, and normalized against tubulin. Protein levels are relative to the initial iDC levels. Mean and SD are shown, n= 3.

Our present study has primarily focused on the LPS induced changes in mRNA levels in moDCs, as we have previously shown that the abundance of an mRNA species generally determines the level of translation to functional protein [8]. However, although transcription and mRNA stability contribute significantly to protein expression, variation in protein concentration is also affected by translational regulation and protein degradation [87]. In order to establish whether the changes in Rab mRNA levels also gave a corresponding change in protein concentration, we evaluated the protein levels for the Rabs that exhibited the largest LPS-induced variation in mRNA expression. Lysates from the same cell cultures used for qPCR were run on SDS gels, blotted onto PVDF membranes and probed with antibodies for Rab7b, Rab27a, Rab21, Rab9, Arf6 or α-tubulin as a loading control, and band intensities were quantified relative to immature moDCs (Figure 7).

We found that immuno-blotting revealed less pronounced responses as compared to qPCR. Although Rab7b, Rab27a and Rab21 all display changes in protein levels somewhat similar to those detected at the mRNA level, the changes are less dramatic, with the 3-4 fold increase in mRNA for Rab7b, Rab27a and Rab21 only producing a 1.2-1.5 increase in protein at 4 hours post LPS (Figure 7B). The level of Arf6, which exhibited a statistically significant increase in mRNA (up 1.8-fold after 4 hours), was not detectably increased. These discrepancies in mRNA and protein levels may be at least in part due to the relatively low resolution of biochemical assays, where small changes in protein expression occur below the threshold for detection. However, the cellular concentrations of mRNA and proteins, as measured by mass spectrometry, generally show a correlation coefficient of only 0.40, implying that translational control and protein degradation contribute significantly to protein levels in the steady state [87]. The dynamic changes in DC function and morphology during maturation make it tempting to speculate that the increased biosynthesis of Rabs could be partly masked by a higher rate of Rab turn-over resulting from adverse effects of GTPase activity or the loss of membranes with which they associate during adhesion, migration, exocytosis or sorting onto internal membranes of multi-vesicular endosomes.

As we could not detect any significant increase in total Arf6 protein concentration by immuno-blotting, we assessed the level of Arf6 activation by a pull-down assay using an effector protein that specifically associates with the active form of Arf6. However, despite an abundant population of active Arf6 in moDCs (as compared to the vitro loaded GTPγS positive control), we could not detect a significant increase after activation (Figure S3). This could be due to the experimental loss of Arf6 containing membranes during harvesting of moDCs, which are predominantly adherent 4 hours after activation, but mostly in suspension before activation and 24 hours after LPS stimulation (Figure S2). However, a study investigating Arf6 in primary murine DCs found that although the GTPase activity of Arf6 was required for LPS-stimulated macropinocytosis and CCL3-induced migration, the fraction of active Arf6 remained constant [88]. This suggests that the apparently consistent concentration of activated and total Arf6 could be due to a higher turn-over through increased use.

## Conclusions

In conclusion, we find that the expression of Rab mRNAs is subject to complex regulation during the LPS-induced maturation of DCs, and that these changes are at least partly reflected at the protein level. We acknowledge that the activity of Rabs depends on their specific GEFs or GAPs, and that effector proteins mediate their functionality. Our study does not address these aspects. However, an altered expression generally implies a change in a cells requirement to achieve a new function. We have attempted to correlate Rab expression profiles with the dynamic changes in DC function through maturation and find that to some extent the increase and decrease of specific Rabs seem to correlate with the altered function of DCs during maturation. The multifaceted roles of Rabs, and the overlapping processes that they regulate, makes it difficult to correlate any specific Rab to a particular process. For example, diverse mechanisms such as phagocytosis, exocytosis and cell migration all require recycling of membranes, and consequently Rabs such as Rab35 and Rab8a have reported roles in all of these processes [14,17,26,89,90]. Clearly we need to develop a better understanding of the intricate roles of Rabs, their diverse and often cell-type specific effectors, and the dynamic membrane sorting events involved in these varied mechanisms. However, with the present approach we have been able to highlight some Rabs, such as Rab7b, Rab21 and Rab27a, which respond strongly to LPS activation and are therefore potent targets for regulating DC specific functions during maturation. Furthermore, our results support the proposed role of transcriptional regulation of Rabs to control cell function during environmentally induced differentiation.

## Methods

### Ethics Statement

Blood components (buffy-coats) from anonymous blood donors were obtained from the local blood bank (Section for Immunology and Blood Transfusion, Ullevål University Hospital, Oslo, Norway) according to the guidelines of the local blood bank approved by the Norwegian Regional Committee for Medical Research Ethics.

### In vitro cell culture and activation of moDCs

Mononuclear cells were isolated from buffy-coats through density gradient centrifugation using Lymphoprep (Axis Shield, Oslo, Norway) followed by negative sorting of monocytes using magnetic beads (MACS-Miltenyi, Auburn, CA, USA). Cells were cultured for 6 days in RPMI (LONZA, Verviers, Belgium) containing 100ng/ml GM-CSF (Immunotools, Friesoythe, Germany), 20ng/ml IL-4 (Invitrogen), 10% FCS (Saveen Werner, Malmö, Uppsala), penicillin/streptomycin and L-Glutamine (BioWhittaker). IL-4 and GM-CFS was replenished every 2 days. After 6 days, the moDCs were activated with 100ng/ml LPS (sc3535, Santa-Cruz Biotechnology, Santa Cruz, CA, USA) or 1xPBS (as negative control). Cells were then harvested at indicated times after activation. For the comparison used in Figure S1, we used HeLa and MelJuSo cell lines cultured in DMEM (LONZA, Verviers, Belgium) supplemented with 10% FCS (Saveen Werner, Malmö, Uppsala), penicillin/streptomycin and L-Glutamine (BioWhittaker).

### Real-time quantitative PCR

Total RNA was isolated from mo-DCs using PerfectPure Cultured Cell Kit (5Prime GmbH, Hamburg, Germany) according to protocols specified by the manufacturer. This includes 15 min. incubation with DNAse to remove genomic DNA before elution of total RNA. 1µg total RNA was used in a 20µl cDNA synthesis reaction using Transcriptor First Strand cDNA Synthesis Kit as described by the manufacturer (Roche Diagnostics, Mannheim, Germany), using oligo dT primers. 300 ng cDNA was used in duplicate real-time PCR reactions using SYBRGreen technology on a LightCycler 480 (Roche), using protocols provided by the manufacturer. Gene-specific primers were created targeting each Rab based on published sequences and compared to previously used, commercially available, primers (Table S2). The relative expression levels of the target genes were calculated using the following formula:

R= (E_target_)^∆Ct target (control-sample)^/(E_reference_)^∆Ct reference (control-sample)^

where E is the primer efficiency E = 10^(-1/slope of standard curve)^. Data was analyzed in Excel (Microsoft) and graphs were made using Prism (GraphPad Software, San Diego, CA, USA). Significance levels were calculated with the Welch’s two-tailed t-test using R (the R Foundation for Statistical Computing).

### Biochemical assays

Day 6 moDCs were seeded out onto 9cm cell culture dishes, stimulated with 100ng/ml LPS for 0, 4, 8, 24 or 48 hours and harvested with a cell scraper on ice, remaining cells were collected by washing out the plates with ice-cold 1xPBS. Cells were spun down at 400rcf for 5 minutes at 4°C and washed in ice cold 1xPBS before centrifugation and aspiration of supernatant. Cells were lysed using a lysis buffer containing 6mM CHAPS, 5mM TrisHCl, 150mM NaCl2, 5mM EDTA, 1% NP40 and protease-arrest (Genotech, St. Louis, MO), and run on SDS-PAGE gels (Pierce, Rockford, IL, USA). Separated proteins were transferred onto PVDF membrane (Millipore, Billerica, MA, USA). For immuno-detection, the following antibodies have been used: anti-tubulin (Zymed, San Francisco, CA, USA), anti-Rab7b (Abnova, Taipei, Taiwan), anti-Rab21 (Sigma-Aldrich, St. Louis, MO, USA), anti-Rab27a, anti-Rab9 and anti-Arf6 (Abcam, Cambridge, UK). HRP-conjugated secondary antibodies were from GE Healthcare. Blots were developed using Amersham ECL Plus Western Blotting Detection System (GE Healthcare). To assess Arf6 activation, cell lysis, pull-down and detection was performed according to the protocol and with reagents supplied by the manufacturer (Cat. # BK033-S, Cytoskeleton, Inc., Denver, CO, USA). Protein levels were quantified by densitometry using Image Quant TL software (GE Healthcare) and normalized against tubulin.

### Microscopy

Day 6 moDCs were stimulated with 100ng/ml LPS for 0, 4 or 24 hours and living cells were imaged on an enclosed Olympus FV1000 system at 37°C, using transmitted light through a UPlan FLN 40x NA 1.30 oil objective (Olympus, Hamburg, Germany).

## Supporting Information

Figure S1 **The expression level of Rab GTPases varies between different cell types.** Quantitative RT-PCR shows expression levels of the targeted Rab GTPases in iDC compared to the cell lines HeLa and MelJuSo. Means and SD are shown on a logarithmic scale, n=3. Expression levels have been normalized to the reference gene GADPH.
(TIF)

Figure S2 **Morphological changes in DCs after LPS stimulation.** Transmission light images of live moDCs stimulated with LPS for the indicated time points. Magnifications of the boxed areas in the top panel are shown in the lower panel.
(TIF)

Figure S3 **Activation of Arf6 during DC maturation.** (A) Immuno-blots showing active (GTP-bound) Arf6 after pull-down, as well as total Arf6 and tubulin (WCL, whole cell lysate), at 0, 4 and 24 hours after LPS stimulation (left panel), or in moDC cell lysates loaded with GTPγS or GDP (right panel, control reactions). (B) Quantification of band intensity by densitometry, normalized against tubulin. Protein levels are relative to the initial iDC levels. Mean and SD are shown, n= 3.
(TIF)

Table S1 **Reference guide to the role of Rab GTPases in important DC functions during the maturation process.** Approximate levels of up or down regulation are also indicated, given in hours after LPS stimulation relative to GADPH or initial Rab expression levels.
(DOCX)

Table S2 **Primer pairs used in qPCR analysis.**
(DOCX)
