# Supplementary figures and images for: Differential Regulation of Rab GTPase Expression in Monocyte-Derived Dendritic Cells upon Lipopolysaccharide Activation: A Correlation to Maturation-Dependent Functional Properties
Source: PLoS One. 2013 Sep 5;8(9):e73538. doi: 10.1371/journal.pone.0073538 (PMC3764041; doi:10.1371/journal.pone.0073538)

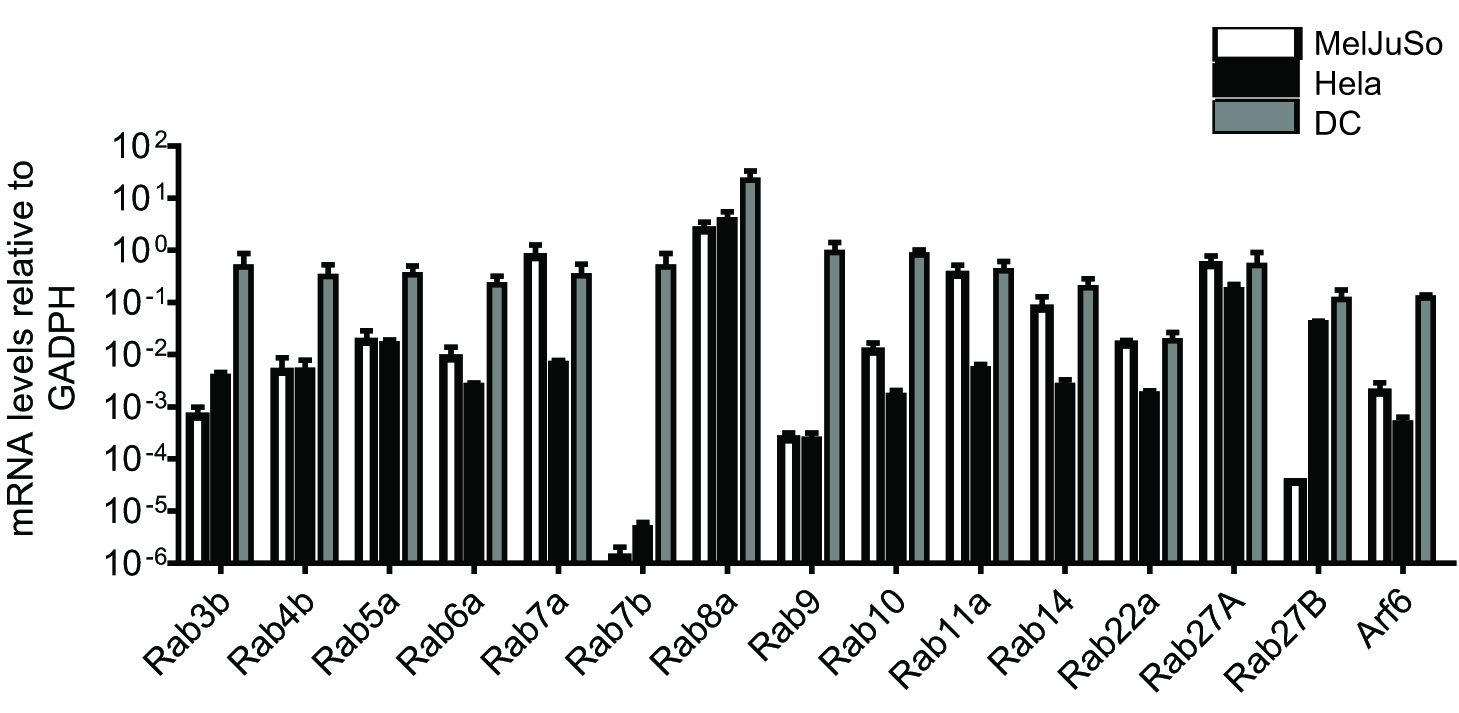

Supplement: Figure S1 — The expression level of Rab GTPases varies between different cell types. Quantitative RT-PCR shows expression levels of the targeted Rab GTPases in iDC compared to the cell lines HeLa and MelJuSo. Means and SD are shown on a logarithmic scale, n=3. Expression levels have been normalized to the reference gene GADPH. (TIF) [file pone.0073538.s001.tif]

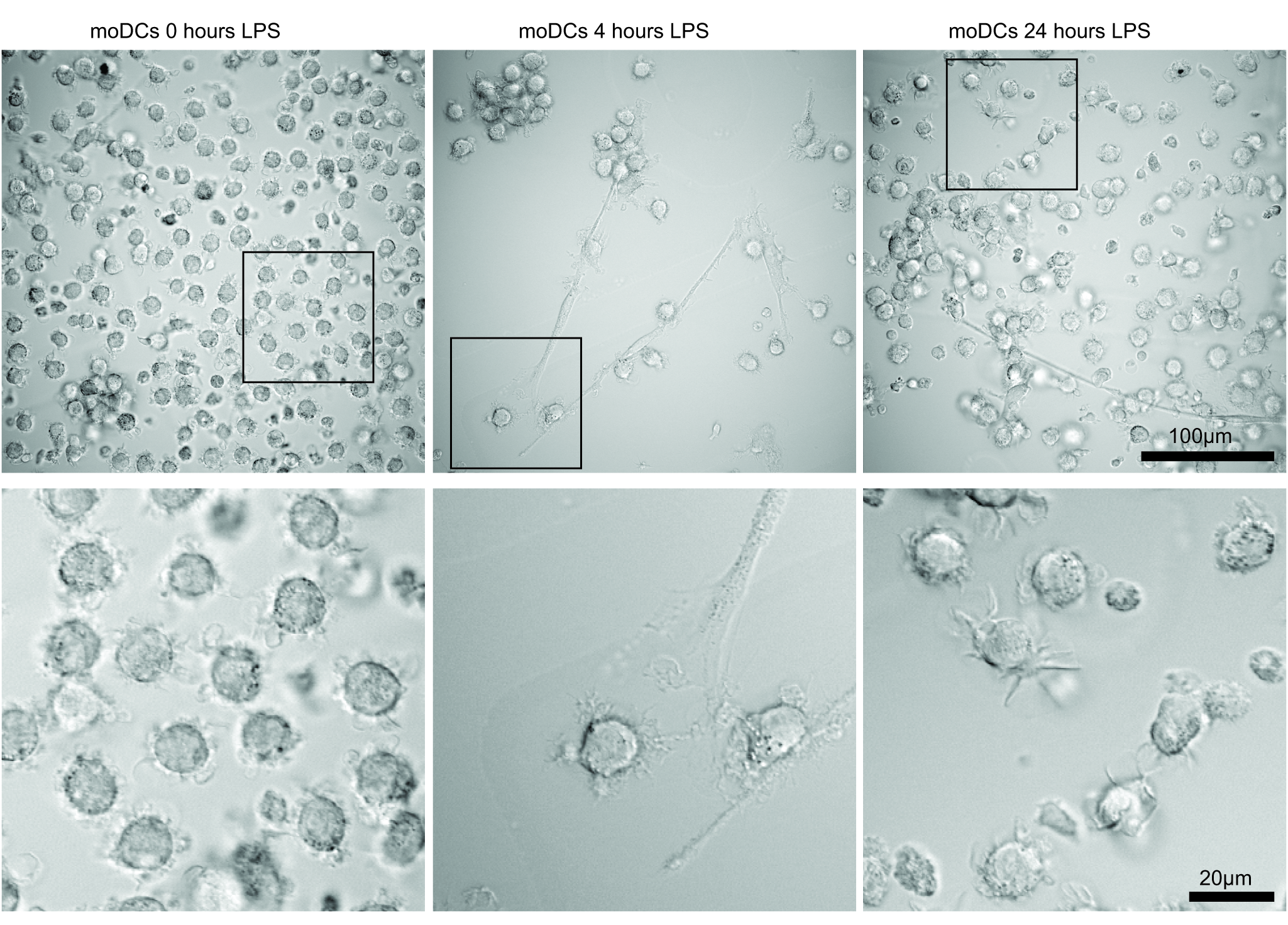

Supplement: Figure S2 — Morphological changes in DCs after LPS stimulation. Transmission light images of live moDCs stimulated with LPS for the indicated time points. Magnifications of the boxed areas in the top panel are shown in the lower panel. (TIF) [file pone.0073538.s002.tif]

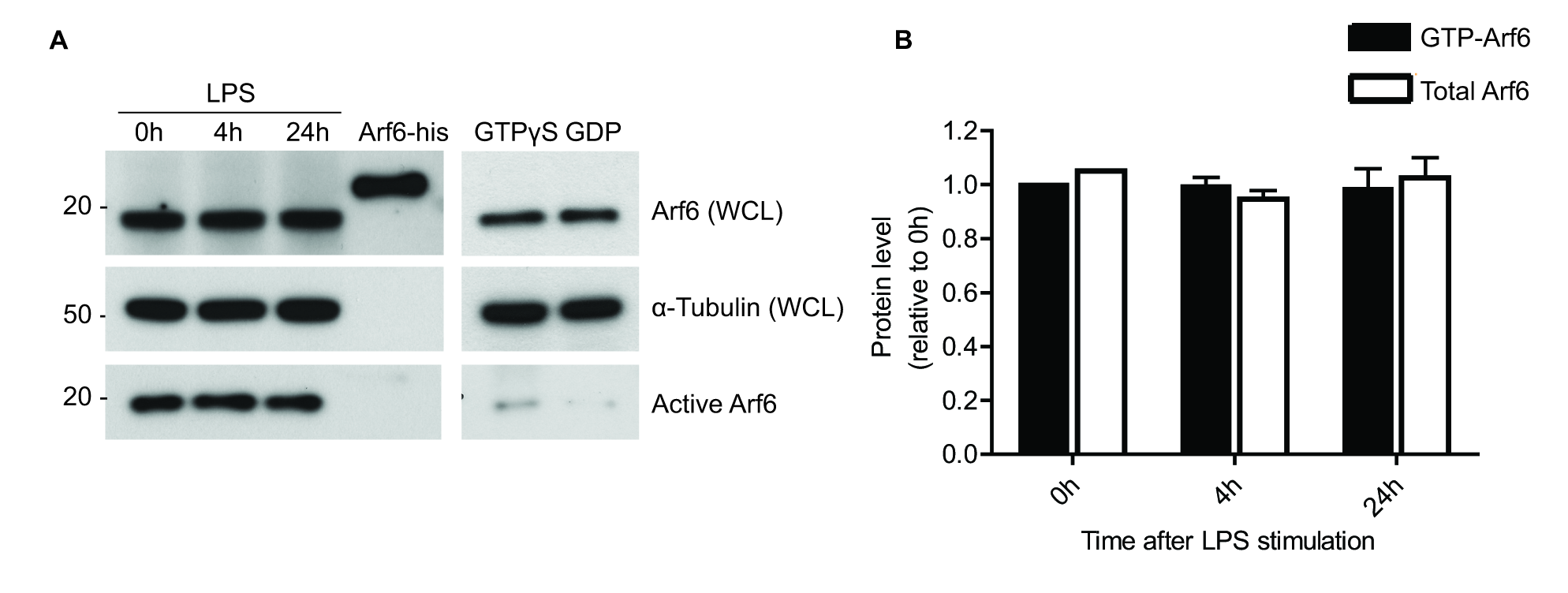

Supplement: Figure S3 — Activation of Arf6 during DC maturation. (A) Immuno-blots showing active (GTP-bound) Arf6 after pull-down, as well as total Arf6 and tubulin (WCL, whole cell lysate), at 0, 4 and 24 hours after LPS stimulation (left panel), or in moDC cell lysates loaded with GTPγS or GDP (right panel, control reactions). (B) Quantification of band intensity by densitometry, normalized against tubulin. Protein levels are relative to the initial iDC levels. Mean and SD are shown, n= 3. (TIF) [file pone.0073538.s003.tif]
