# Supplementary material for: Differential Regulation of Rab GTPase Expression in Monocyte-Derived Dendritic Cells upon Lipopolysaccharide Activation: A Correlation to Maturation-Dependent Functional Properties
Source: PLoS One. 2013 Sep 5;8(9):e73538. doi: 10.1371/journal.pone.0073538 (PMC3764041; doi:10.1371/journal.pone.0073538)
Supplement: Table S1 — Reference guide to the role of Rab GTPases in important DC functions during the maturation process. Approximate levels of up or down regulation are also indicated, given in hours after LPS stimulation relative to GADPH or initial Rab expression levels. (DOCX) [file pone.0073538.s004.docx]

| **GTPase** | **Rab mRNA levels*** | | | **Rab function references** | | | | | |
| --- | --- | --- | --- | --- | --- | --- | --- | --- | --- |
|  | **0^$^** | **4-8^§^** | **24-48^§^** | **Macropinocytosis** | **Phagocytosis** | **Endosomal recycling** | **Endosomal maturation** | **Secretion/exocytosis** | **Adhesion/extension/migration** |
| Arf6 | 0.2 | ∧ 1.8 | ∨ 0.5 | Rac activation/actin polymerization[[1](#_ENREF_1), [2](#_ENREF_2)] | Actin remodeling[[3](#_ENREF_3)] | With Rab35 in MHC II recycling[[4](#_ENREF_4)], negatively regulates Rab4 recycling[[5](#_ENREF_5)], with Rab8a and Rab35 coordinates recycling of secretory vesicles[[6](#_ENREF_6)] |  |  | Via Arap2 (Arf-GAP) and rac regulates focal adhesion[[7](#_ENREF_7)], with Rab35 recycling in cell adhesion[[8](#_ENREF_8)] |
| Rab3b | 0.3 | ∧ 1.3 | ∨ 0.9 |  |  | Fast recycling cross-presentation in DCs[[9](#_ENREF_9)] |  | Calcium induced exocytosis[[10](#_ENREF_10)], secretion of TNF-α[[11](#_ENREF_11)],  WPB exocytosis[[12](#_ENREF_12)] |  |
| Rab4b | 0.3 | ∧ 1.5 | ∧ 1.2 |  |  | Rapid recycling[[13](#_ENREF_13), [14](#_ENREF_14)], in B cell-, Fc-receptor antigen presentation[[15](#_ENREF_15)] |  |  | 1. recycling endosomes, integrin recycling[[16](#_ENREF_16)] |
| Rab5 | 0.3 | ∧ 1.5 | ∨ 0.8 | Actin remodeling[[17](#_ENREF_17)], APPL1 interaction[[18](#_ENREF_18), [19](#_ENREF_19)], rate of formation[[20](#_ENREF_20)] |  |  | Biogenesis of endocytic pathway[[21](#_ENREF_21)] |  | Integrin transport[[22](#_ENREF_22)], rac transport[[23](#_ENREF_23)] |
| Rab6a | 0.2 | ∧ 1.8 | 1.0 |  |  |  |  | Targeting of exocytic carriers[[24](#_ENREF_24)], Golgi to cell surface transport[[25](#_ENREF_25)], TNF secretion[[26](#_ENREF_26)] |  |
| Rab7a | 0.3 | ∨ 0.8 | ∨ 0.5 |  | Phagosomal maturation[[27](#_ENREF_27), [28](#_ENREF_28)] |  | Biogenesis, fusion, transport[[29](#_ENREF_29)] |  |  |
| Rab7b | 0.5 | ∧ 3.5 | ∨ 0.9 |  |  | Late endosome to Golgi recycling of receptors[[30](#_ENREF_30), [31](#_ENREF_31)] | Reduced levels of TLR4/9[[32](#_ENREF_32), [33](#_ENREF_33)], Cathapsin D maturation[[31](#_ENREF_31)] |  |  |
| Rab8a | 22.0 | ∧ 1.5 | ∧ 1.4 | Promotes protrusion[[34](#_ENREF_34)] |  | With Arf6 and Rab35 coordinates recycling and secretory vesicles[[6](#_ENREF_6)] | Actin based movement of lysosome related organelles[[35](#_ENREF_35)] | Docking and fusion of exocytic vesicles[[36](#_ENREF_36)], basolateral secretion[[37](#_ENREF_37)], GLUT4 secretion[[38](#_ENREF_38)] | Extension of membrane[[39](#_ENREF_39)], with rab11[[40](#_ENREF_40)] |
| Rab9 | 0.9 | 1.0 | 1.0 |  |  | Late endosome to Golgi recycling of M6PR[[41](#_ENREF_41)], [[42](#_ENREF_42)] | Biogenesis/M6PR recycling[[41](#_ENREF_41), [43](#_ENREF_43)] |  |  |
| Rab10 | 0.8 | ∧ 1.3 | ∨ 0.3 |  |  | With Arf6 on recycling endosomes[[44](#_ENREF_44)] | Phagosomal maturation[[45](#_ENREF_45)] | Replenish TLR4 from Golgi to cell surface[[46](#_ENREF_46)], docking of GLUT4 vesicles[[47](#_ENREF_47)], ER morphology[[48](#_ENREF_48)] |  |
| Rab11a | 0.4 | ∧1.4 | ∨ 0.8 |  | Recycling to enhance phagocytosis[[49](#_ENREF_49)] | Cell surface recycling[[50](#_ENREF_50), [51](#_ENREF_51)] |  | FcRn exocytosis [[52](#_ENREF_52)] | Integrin recycling[[53](#_ENREF_53)], extension of membrane with rab8a[[40](#_ENREF_40)] |
| Rab14 | 0.2 | ∨ 0.7 | ∨ 0.4 |  |  | Transferrin receptor recycling [[54](#_ENREF_54)], recycling to regulate cell-cell junction[[55](#_ENREF_55)] | Prevents phagosomal maturation[[56](#_ENREF_56)], Golgi to endosome transport[[57](#_ENREF_57)], IRAP transport for cross-presentation[[58](#_ENREF_58)] |  |  |
| Rab21 | 0.02 | ∧ 3.8 | ∧ 1.2 | Present on macropinosomes[[59](#_ENREF_59)]  APPL1 interaction[[18](#_ENREF_18), [19](#_ENREF_19)] |  |  | Present on macropinosomes[[59](#_ENREF_59)], Defect in endocytosis[[60](#_ENREF_60)]  Endosomal dynamics[[61](#_ENREF_61), [62](#_ENREF_62)] |  | Integrin transport, adhesion/motility[[22](#_ENREF_22), [63](#_ENREF_63)], membrane extension via Varp (Rab21-GEF)[ [64](#_ENREF_64), [65](#_ENREF_65)] |
| Rab22a | 0.5 | ∨ 0.5 | ∨ 0.4 |  |  | Formation of tubular recycling endosomes for cell surface recycling[[66](#_ENREF_66)] | Prevent phagosomal maturation[[67](#_ENREF_67)], regulates early endosome dynamics with Rabex5[[68](#_ENREF_68)] |  |  |
| Rab27a | 0.1 | ∧ 3.0 | ∧ 2.5 |  | Reduced phagosomal degradation, pH regulation[[69](#_ENREF_69)] |  |  | Loss of cytotoxic killing[[70](#_ENREF_70)], secretory lysosome[[71](#_ENREF_71)], secretion of granules[[72](#_ENREF_72), [73](#_ENREF_73)], exosome secretion[[74](#_ENREF_74)], regulated exocytosis[[75](#_ENREF_75)] |  |
| Rab27b | 0.01 | ∨ 0.6 | ∨ 0.8 |  |  |  |  | Secretion of granules[[76](#_ENREF_76), [77](#_ENREF_77)] |  |
| Rab35 | 0.1 | ∧ 1.4 | ∨ 0.7 |  | Rac and Cdc42 recruitment[[78](#_ENREF_78)], recruits ACAP2 (Arf6-GAP) to promote phagocytosis[[79](#_ENREF_79)] | With Arf6 in MHC II recycling[[4](#_ENREF_4)], with Arf6 to regulate cell adhesion/recycling[[8](#_ENREF_8)], with Arf6 and Rab8a coordinate recycling of secretory vesicles[[6](#_ENREF_6)], TcR recycling[[80](#_ENREF_80)] |  | Exosome secretion[[81](#_ENREF_81)] | Actin assembly via fascin[[82](#_ENREF_82)], membrane extension via cdc42[[83](#_ENREF_83)], via ACAP2 (arf6-GAP)[ [84](#_ENREF_84)], with Arf6 to regulate cell adhesion/recycling[[8](#_ENREF_8)] |
| *Hours after LPS, ^$^Relative to GAPDH, ^§^Relative to immature levels | | | | | | | | | |

**Table S1: Reference guide to the role of Rab GTPases in important DC functions during the maturation process.** Approximate levels of up or down regulation are also indicated, given in hours after LPS stimulation relative to GADPH or initial Rab expression levels.

References:

1. Zhang Q, Calafat J, Janssen H, Greenberg S (1999) ARF6 is required for growth factor- and rac-mediated membrane ruffling in macrophages at a stage distal to rac membrane targeting. Mol Cell Biol 19: 8158-8168.

2. Radhakrishna H, Al-Awar O, Khachikian Z, Donaldson JG (1999) ARF6 requirement for Rac ruffling suggests a role for membrane trafficking in cortical actin rearrangements. J Cell Sci 112 ( Pt 6): 855-866.

3. Zhang Q, Cox D, Tseng CC, Donaldson JG, Greenberg S (1998) A requirement for ARF6 in Fcgamma receptor-mediated phagocytosis in macrophages. J Biol Chem 273: 19977-19981.

4. Walseng E, Bakke O, Roche PA (2008) Major histocompatibility complex class II-peptide complexes internalize using a clathrin- and dynamin-independent endocytosis pathway. J Biol Chem 283: 14717-14727.

5. Macia E, Partisani M, Paleotti O, Luton F, Franco M (2012) Arf6 negatively controls the rapid recycling of the beta2 adrenergic receptor. J Cell Sci 125: 4026-4035.

6. Rahajeng J, Giridharan SS, Cai B, Naslavsky N, Caplan S (2012) MICAL-L1 is a tubular endosomal membrane hub that connects Rab35 and Arf6 with Rab8a. Traffic 13: 82-93.

7. Chen PW, Jian X, Yoon HY, Randazzo PA (2013) ARAP2 signals through Arf6 and Rac1 to control focal adhesion morphology. J Biol Chem.

8. Allaire PD, Seyed Sadr M, Chaineau M, Seyed Sadr E, Konefal S, et al. (2012) Interplay between Rab35 and Arf6 controls cargo recycling to coordinate cell adhesion and recycling. J Cell Sci.

9. Zou L, Zhou J, Zhang J, Li J, Liu N, et al. (2009) The GTPase Rab3b/3c-positive recycling vesicles are involved in cross-presentation in dendritic cells. Proc Natl Acad Sci U S A 106: 15801-15806.

10. Lledo PM, Vernier P, Vincent JD, Mason WT, Zorec R (1993) Inhibition of Rab3B expression attenuates Ca(2+)-dependent exocytosis in rat anterior pituitary cells. Nature 364: 540-544.

11. Mori R, Ikematsu K, Kitaguchi T, Kim SE, Okamoto M, et al. (2011) Release of TNF-alpha from macrophages is mediated by small GTPase Rab37. Eur J Immunol 41: 3230-3239.

12. Bierings R, Hellen N, Kiskin N, Knipe L, Fonseca AV, et al. (2012) The interplay between the Rab27A effectors Slp4-a and MyRIP controls hormone-evoked Weibel-Palade body exocytosis. Blood 120: 2757-2767.

13. van der Sluijs P, Hull M, Webster P, Male P, Goud B, et al. (1992) The small GTP-binding protein rab4 controls an early sorting event on the endocytic pathway. Cell 70: 729-740.

14. Novick P, Zerial M (1997) The diversity of Rab proteins in vesicle transport. Curr Opin Cell Biol 9: 496-504.

15. Lazzarino DA, Blier P, Mellman I (1998) The monomeric guanosine triphosphatase rab4 controls an essential step on the pathway of receptor-mediated antigen processing in B cells. J Exp Med 188: 1769-1774.

16. Roberts M, Barry S, Woods A, van der Sluijs P, Norman J (2001) PDGF-regulated rab4-dependent recycling of alphavbeta3 integrin from early endosomes is necessary for cell adhesion and spreading. Curr Biol 11: 1392-1402.

17. Lanzetti L, Palamidessi A, Areces L, Scita G, Di Fiore PP (2004) Rab5 is a signalling GTPase involved in actin remodelling by receptor tyrosine kinases. Nature 429: 309-314.

18. Zhu G, Chen J, Liu J, Brunzelle JS, Huang B, et al. (2007) Structure of the APPL1 BAR-PH domain and characterization of its interaction with Rab5. Embo J 26: 3484-3493.

19. Zoncu R, Perera RM, Balkin DM, Pirruccello M, Toomre D, et al. (2009) A phosphoinositide switch controls the maturation and signaling properties of APPL endosomes. Cell 136: 1110-1121.

20. Li G, D'Souza-Schorey C, Barbieri MA, Cooper JA, Stahl PD (1997) Uncoupling of membrane ruffling and pinocytosis during Ras signal transduction. J Biol Chem 272: 10337-10340.

21. Zeigerer A, Gilleron J, Bogorad RL, Marsico G, Nonaka H, et al. (2012) Rab5 is necessary for the biogenesis of the endolysosomal system in vivo. Nature 485: 465-470.

22. Pellinen T, Arjonen A, Vuoriluoto K, Kallio K, Fransen JA, et al. (2006) Small GTPase Rab21 regulates cell adhesion and controls endosomal traffic of beta1-integrins. J Cell Biol 173: 767-780.

23. Palamidessi A, Frittoli E, Garre M, Faretta M, Mione M, et al. (2008) Endocytic trafficking of Rac is required for the spatial restriction of signaling in cell migration. Cell 134: 135-147.

24. Grigoriev I, Splinter D, Keijzer N, Wulf PS, Demmers J, et al. (2007) Rab6 regulates transport and targeting of exocytotic carriers. Dev Cell 13: 305-314.

25. Storrie B, Micaroni M, Morgan GP, Jones N, Kamykowski JA, et al. (2012) Electron tomography reveals Rab6 is essential to the trafficking of trans-Golgi clathrin and COPI-coated vesicles and the maintenance of Golgi cisternal number. Traffic 13: 727-744.

26. Micaroni M, Stanley AC, Khromykh T, Venturato J, Wong CXF, et al. (2013) Rab6a/a’ Are Important Golgi Regulators of Pro-Inflammatory TNF Secretion in Macrophages. PLoS ONE 8: e57034.

27. Harrison RE, Bucci C, Vieira OV, Schroer TA, Grinstein S (2003) Phagosomes Fuse with Late Endosomes and/or Lysosomes by Extension of Membrane Protrusions along Microtubules: Role of Rab7 and RILP. Molecular and Cellular Biology 23: 6494-6506.

28. Vieira OV, Bucci C, Harrison RE, Trimble WS, Lanzetti L, et al. (2003) Modulation of Rab5 and Rab7 Recruitment to Phagosomes by Phosphatidylinositol 3-Kinase. Molecular and Cellular Biology 23: 2501-2514.

29. Wang T, Ming Z, Xiaochun W, Hong W (2011) Rab7: role of its protein interaction cascades in endo-lysosomal traffic. Cell Signal 23: 516-521.

30. Bucci C, Bakke O, Progida C (2010) Rab7b and receptors trafficking. Commun Integr Biol 3: 401-404.

31. Progida C, Cogli L, Piro F, De Luca A, Bakke O, et al. (2010) Rab7b controls trafficking from endosomes to the TGN. J Cell Sci 123: 1480-1491.

32. Yao M, Liu X, Li D, Chen T, Cai Z, et al. (2009) Late endosome/lysosome-localized Rab7b suppresses TLR9-initiated proinflammatory cytokine and type I IFN production in macrophages. J Immunol 183: 1751-1758.

33. Wang Y, Chen T, Han C, He D, Liu H, et al. (2007) Lysosome-associated small Rab GTPase Rab7b negatively regulates TLR4 signaling in macrophages by promoting lysosomal degradation of TLR4. Blood 110: 962-971.

34. Hattula K, Furuhjelm J, Tikkanen J, Tanhuanpaa K, Laakkonen P, et al. (2006) Characterization of the Rab8-specific membrane traffic route linked to protrusion formation. J Cell Sci 119: 4866-4877.

35. Chabrillat ML, Wilhelm C, Wasmeier C, Sviderskaya EV, Louvard D, et al. (2005) Rab8 regulates the actin-based movement of melanosomes. Mol Biol Cell 16: 1640-1650.

36. Grigoriev I, Yu Ka L, Martinez-Sanchez E, Serra-Marques A, Smal I, et al. (2011) Rab6, Rab8, and MICAL3 Cooperate in Controlling Docking and Fusion of Exocytotic Carriers. Current Biology 21: 967-974.

37. Henry L, Sheff DR (2008) Rab8 regulates basolateral secretory, but not recycling, traffic at the recycling endosome. Mol Biol Cell 19: 2059-2068.

38. Sun Y, Bilan PJ, Liu Z, Klip A (2010) Rab8A and Rab13 are activated by insulin and regulate GLUT4 translocation in muscle cells. Proc Natl Acad Sci U S A 107: 19909-19914.

39. Nachury MV, Loktev AV, Zhang Q, Westlake CJ, Peranen J, et al. (2007) A core complex of BBS proteins cooperates with the GTPase Rab8 to promote ciliary membrane biogenesis. Cell 129: 1201-1213.

40. Knodler A, Feng S, Zhang J, Zhang X, Das A, et al. (2010) Coordination of Rab8 and Rab11 in primary ciliogenesis. Proc Natl Acad Sci U S A 107: 6346-6351.

41. Lombardi D, Soldati T, Riederer MA, Goda Y, Zerial M, et al. (1993) Rab9 functions in transport between late endosomes and the trans Golgi network. Embo J 12: 677-682.

42. Carroll KS, Hanna J, Simon I, Krise J, Barbero P, et al. (2001) Role of Rab9 GTPase in facilitating receptor recruitment by TIP47. Science 292: 1373-1376.

43. Riederer MA, Soldati T, Shapiro AD, Lin J, Pfeffer SR (1994) Lysosome biogenesis requires Rab9 function and receptor recycling from endosomes to the trans-Golgi network. J Cell Biol 125: 573-582.

44. Shi A, Liu O, Koenig S, Banerjee R, Chen CC-H, et al. (2012) RAB-10-GTPase–mediated regulation of endosomal phosphatidylinositol-4,5-bisphosphate. Proceedings of the National Academy of Sciences 109: E2306–E2315.

45. Cardoso CM, Jordao L, Vieira OV (2010) Rab10 regulates phagosome maturation and its overexpression rescues Mycobacterium-containing phagosomes maturation. Traffic 11: 221-235.

46. Wang D, Lou J, Ouyang C, Chen W, Liu Y, et al. (2010) Ras-related protein Rab10 facilitates TLR4 signaling by promoting replenishment of TLR4 onto the plasma membrane. Proc Natl Acad Sci U S A 107: 13806-13811.

47. Chen Y, Wang Y, Zhang J, Deng Y, Jiang L, et al. (2012) Rab10 and myosin-Va mediate insulin-stimulated GLUT4 storage vesicle translocation in adipocytes. J Cell Biol 198: 545-560.

48. English AR, Voeltz GK (2013) Rab10 GTPase regulates ER dynamics and morphology. Nat Cell Biol 15: 169-178.

49. Cox D, Lee DJ, Dale BM, Calafat J, Greenberg S (2000) A Rab11-containing rapidly recycling compartment in macrophages that promotes phagocytosis. Proc Natl Acad Sci U S A 97: 680-685.

50. Ullrich O, Reinsch S, Urbe S, Zerial M, Parton RG (1996) Rab11 regulates recycling through the pericentriolar recycling endosome. J Cell Biol 135: 913-924.

51. Jones MC, Caswell PT, Norman JC (2006) Endocytic recycling pathways: emerging regulators of cell migration. Curr Opin Cell Biol 18: 549-557.

52. Ward ES, Martinez C, Vaccaro C, Zhou J, Tang Q, et al. (2005) From sorting endosomes to exocytosis: association of Rab4 and Rab11 GTPases with the Fc receptor, FcRn, during recycling. Mol Biol Cell 16: 2028-2038.

53. Caswell PT, Norman JC (2006) Integrin trafficking and the control of cell migration. Traffic 7: 14-21.

54. Yamamoto H, Koga H, Katoh Y, Takahashi S, Nakayama K, et al. (2010) Functional cross-talk between Rab14 and Rab4 through a dual effector, RUFY1/Rabip4. Mol Biol Cell 21: 2746-2755.

55. Linford A, Yoshimura S, Nunes Bastos R, Langemeyer L, Gerondopoulos A, et al. (2012) Rab14 and its exchange factor FAM116 link endocytic recycling and adherens junction stability in migrating cells. Dev Cell 22: 952-966.

56. Kyei GB, Vergne I, Chua J, Roberts E, Harris J, et al. (2006) Rab14 is critical for maintenance of Mycobacterium tuberculosis phagosome maturation arrest. Embo J 25: 5250-5259.

57. Junutula JR, De Maziere AM, Peden AA, Ervin KE, Advani RJ, et al. (2004) Rab14 is involved in membrane trafficking between the Golgi complex and endosomes. Mol Biol Cell 15: 2218-2229.

58. Weimershaus M, Maschalidi S, Sepulveda F, Manoury B, van Endert P, et al. (2012) Conventional dendritic cells require IRAP-Rab14 endosomes for efficient cross-presentation. J Immunol 188: 1840-1846.

59. Egami Y, Araki N (2009) Dynamic changes in the spatiotemporal localization of Rab21 in live RAW264 cells during macropinocytosis. PLoS One 4: e6689.

60. Simpson JC, Griffiths G, Wessling-Resnick M, Fransen JA, Bennett H, et al. (2004) A role for the small GTPase Rab21 in the early endocytic pathway. J Cell Sci 117: 6297-6311.

61. Zhang X, He X, Fu XY, Chang Z (2006) Varp is a Rab21 guanine nucleotide exchange factor and regulates endosome dynamics. J Cell Sci 119: 1053-1062.

62. Delprato A, Merithew E, Lambright DG (2004) Structure, exchange determinants, and family-wide rab specificity of the tandem helical bundle and Vps9 domains of Rabex-5. Cell 118: 607-617.

63. Hooper S, Gaggioli C, Sahai E (2010) A chemical biology screen reveals a role for Rab21-mediated control of actomyosin contractility in fibroblast-driven cancer invasion. Br J Cancer 102: 392-402.

64. Burgo A, Sotirakis E, Simmler MC, Verraes A, Chamot C, et al. (2009) Role of Varp, a Rab21 exchange factor and TI-VAMP/VAMP7 partner, in neurite growth. EMBO Rep 10: 1117-1124.

65. Ohbayashi N, Yatsu A, Tamura K, Fukuda M (2012) The Rab21-GEF activity of Varp, but not its Rab32/38 effector function, is required for dendrite formation in melanocytes. Mol Biol Cell 23: 669-678.

66. Weigert R, Yeung AC, Li J, Donaldson JG (2004) Rab22a regulates the recycling of membrane proteins internalized independently of clathrin. Mol Biol Cell 15: 3758-3770.

67. Roberts EA, Chua J, Kyei GB, Deretic V (2006) Higher order Rab programming in phagolysosome biogenesis. J Cell Biol 174: 923-929.

68. Zhu H, Liang Z, Li G (2009) Rabex-5 Is a Rab22 Effector and Mediates a Rab22-Rab5 Signaling Cascade in Endocytosis. Molecular Biology of the Cell 20: 4720-4729.

69. Jancic C, Savina A, Wasmeier C, Tolmachova T, El-Benna J, et al. (2007) Rab27a regulates phagosomal pH and NADPH oxidase recruitment to dendritic cell phagosomes. Nat Cell Biol 9: 367-378.

70. Fukuda M (2005) Versatile role of Rab27 in membrane trafficking: focus on the Rab27 effector families. J Biochem 137: 9-16.

71. Stinchcombe J, Bossi G, Griffiths GM (2004) Linking albinism and immunity: the secrets of secretory lysosomes. Science 305: 55-59.

72. Herrero-Turrion MJ, Calafat J, Janssen H, Fukuda M, Mollinedo F (2008) Rab27a regulates exocytosis of tertiary and specific granules in human neutrophils. J Immunol 181: 3793-3803.

73. Catz SD (2013) Regulation of vesicular trafficking and leukocyte function by Rab27 GTPases and their effectors. J Leukoc Biol.

74. Ostrowski M, Carmo NB, Krumeich S, Fanget I, Raposo G, et al. (2010) Rab27a and Rab27b control different steps of the exosome secretion pathway. Nat Cell Biol 12: 19-30; sup pp 11-13.

75. Tolmachova T, Anders R, Stinchcombe J, Bossi G, Griffiths GM, et al. (2004) A general role for Rab27a in secretory cells. Mol Biol Cell 15: 332-344.

76. Mizuno K, Tolmachova T, Ushakov DS, Romao M, Abrink M, et al. (2007) Rab27b regulates mast cell granule dynamics and secretion. Traffic 8: 883-892.

77. Shirakawa R, Higashi T, Tabuchi A, Yoshioka A, Nishioka H, et al. (2004) Munc13-4 is a GTP-Rab27-binding protein regulating dense core granule secretion in platelets. J Biol Chem 279: 10730-10737.

78. Shim J, Lee SM, Lee MS, Yoon J, Kweon HS, et al. (2010) Rab35 mediates transport of Cdc42 and Rac1 to the plasma membrane during phagocytosis. Mol Cell Biol 30: 1421-1433.

79. Egami Y, Fukuda M, Araki N (2011) Rab35 regulates phagosome formation through recruitment of ACAP2 in macrophages during FcgammaR-mediated phagocytosis. J Cell Sci 124: 3557-3567.

80. Patino-Lopez G, Dong X, Ben-Aissa K, Bernot KM, Itoh T, et al. (2008) Rab35 and its GAP EPI64C in T cells regulate receptor recycling and immunological synapse formation. J Biol Chem 283: 18323-18330.

81. Hsu C, Morohashi Y, Yoshimura S, Manrique-Hoyos N, Jung S, et al. (2010) Regulation of exosome secretion by Rab35 and its GTPase-activating proteins TBC1D10A-C. J Cell Biol 189: 223-232.

82. Zhang J, Fonovic M, Suyama K, Bogyo M, Scott MP (2009) Rab35 controls actin bundling by recruiting fascin as an effector protein. Science 325: 1250-1254.

83. Chevallier J, Koop C, Srivastava A, Petrie RJ, Lamarche-Vane N, et al. (2009) Rab35 regulates neurite outgrowth and cell shape. FEBS Lett 583: 1096-1101.

84. Kobayashi H, Fukuda M (2012) Rab35 regulates Arf6 activity through centaurin-beta2 (ACAP2) during neurite outgrowth. J Cell Sci 125: 2235-2243.
