# Supplementary material for: Differential Regulation of Rab GTPase Expression in Monocyte-Derived Dendritic Cells upon Lipopolysaccharide Activation: A Correlation to Maturation-Dependent Functional Properties
Source: PLoS One. 2013 Sep 5;8(9):e73538. doi: 10.1371/journal.pone.0073538 (PMC3764041; doi:10.1371/journal.pone.0073538)
Supplement: Table S2 — Primer pairs used in qPCR analysis. (DOCX) [file pone.0073538.s005.docx]

**Table S2: Primer pairs used in qPCR analysis**

| Target gene | qPCR primer pair |
| --- | --- |
| Arf6 | F: CCAAGGTCTCATCTTCGTAGTGG R: AGGTCCTGCTTGTTGGCGAAGA |
| Rab3b | F: CGGACCATCACAACAGCCTATT  R: GTAGCCCAGTCTTGGACAGCAT |
| Rab4b | F: CCTCCCGCTCAGGGTCCAG R: GGCTGCCAGCCGAGGAGC |
| Rab5 | F: ACTTCTGGGAGAGTCCGCTGTT R: GTGTCATCAAGACATACAGTTTGG |
| Rab6 | F: CTCTTTCGACGTGTAGCAGCAG R: AGGAACAGCCTCCTTCACTGAC |
| Rab7a | F: AGTCTGCACCTCTGTAGAAGGC R: GTGATGGTGGATGACAGGCTAG |
| Rab7b | F: GGCCAGCATCCTCTCCAAGATTATC R: GATGCAGCCATCGGAGCCCTTGT |
| Rab8a | F: TCAGGAACGGTTTCGGACCGATC R: GCTCCTCAATGTTGCGAATCCAG |
| Rab9 | F: AATAATTCTTCTTGGAGATGGTGGAGTTG R: ACTAAATGTAAGCAGGCAACAGTCAGAAC |
| Rab10 | F: AAGGCGTTCCTCACGTTAGCTG  R: GGAACAGGAGAATGCTCAGCAG |
| Rab11a | F: AGCACCATTGGAGTAGAGTTTGC  R: AAGGCACCTACAGCTCCACGAT |
| Rab14 | F: GCGATTTAGGGCTGTTACACGG  R: CCTTGCATCTGTCAACCAGCTG |
| Rab21 | F: CGACAAGCACATCACCACTCTG  R: GCATGGAATCTCTCTTGACCTGC |
| Rab22a | F: GCACCAATGTACTATCGAGGGTC  R: CATGCTGTCGAAGCTCTTTCACC |
| Rab27a | F: GAAGCCATAGCACTCGCAGAGA  R: CAGGACTTGTCCACACACCGTT |
| Rab27b | F: CCTCACCAGTCAACAGAGCTTC  R: GCCGTTCATTGACTTCCCTTTGG |
| Rab35 | F: CAGCCCATCTTACTGCAAGCAG  R: GCTGACAACCTGTCGGAGAGAA |
| GADPH | F: CCACATCGCTCAGACACCAT  R: GGCAACAATATTCCACTTTACCAGAGT |
